# The efficacy of Er,Cr:YSGG laser supported periodontal therapy on the reduction of peridodontal disease related oral malodor: a randomized clinical study

**DOI:** 10.1186/s13005-016-0116-y

**Published:** 2016-05-04

**Authors:** Ömür Dereci, Mükerrem Hatipoğlu, Alper Sindel, Sinan Tozoğlu, Kemal Üstün

**Affiliations:** Department of Oral and Maxillofacial Surgery, Faculty of Dentistry, Eskişehir Osmangazi University, Meşelik Campus, 26480 Eskişehir, Turkey; Department of Periodontology, Faculty of Dentistry, Akdeniz University, Antalya, Turkey; Department of Oral and Maxillofacial Surgery, Faculty of Dentistry, Akdeniz University, Antalya, Turkey

**Keywords:** Halitosis, Periodontitis, Subgingival curettage, YSGG laser

## Abstract

**Background:**

This study aims to evaluate the efficacy of Er,Cr:YSGG laser assisted periodontal therapy on the reduction of oral malodor and periodontal disease.

**Methods:**

Sixty patients with chronic periodontitis were included in the study and allocated into two groups each containing 30 patients. The study was planned in a double blind fashion. Conventional periodontal therapy was performed in group 1 and conventional periodontal therapy was performed in association with Er,Cr:YSGG application in group 2. Periodontal parameters of probing depth, clinical attachment level, plaque index and bleeding on probing were measured with a periodontal probe. Quantitative analysis of volatile sulphure compunds (VSCs) were measured with a calibrated halimeter at baseline level and at post-treatment 1st, 3rd and 6th months. *P* values <0.05 were accepted as statistically significant.

**Results:**

There was a statistical significant reduction in VSC values in group 2 at post-treatment 3rd and 6th months (*p* < 0.05). Pocket depth values at post-treatment 1st month and bleeding on probing values at post-treatment 3rd and 6th months were significantly decreased in group 2 (*p* < 0.05). Intragroup statistical analysis revealed that there were statistically significant differences for all parameters (*p* < 0.01).

**Conclusions:**

Er,Cr:YSGG laser assisted conventional periodontal therapy is more effective in reducing oral malodor and improving periodontal healing compared to conventional periodontal therapy alone.

## Background

Periodontal treatment aims to reduce and remove periodontal diseases with a variety of techniques such as scaling and root plaining and flap procedures [1, 2]. Mechanical therapy of periodontal diseases have been applied securely with high success rates for years. The improvement in bacterial reduction in periodontal pocket and clinical healing appeared to be better in patients with conventional periodontal therapy assisted with adjunctive therapy than in patients with conventional periodontal therapy alone [2, 3].

Lately, there have been several improvements in the treatment of periodontal disease, such as the usage of hard and soft lasers as an adjunctive to conventional periodontal therapy for the effective reduction and elimination of pathogenic microorganisms in the periodontal pocket, thus, leading to a more effective and pain free treatment [3–7]. As a member of the erbium laser family, Er,Cr:YSGG lasers demonstrate a very shallow penetration in tissue with a wavelength of 2.78 μm posing minimal thermal risk to the deeper tissues when compared with other lasers and provide a better surface for the attachment of blood derived components on roots [8, 9]. It is also rerported that ER,Cr,YSGG laser enhances cell attachment and migration on the root surfaces [10]. The morphological surface alterations promoted by Er,Cr:YSGG have been related to its high absorption in water [11, 12]. The utilization of Er,Cr:YSGG laser adjunctive to conventional periodontal therapy is reported to be more effective in bacterial reduction compared to conventional periodontal therapy [1, 8]. In addition to the bacterial reduction, Er,Cr:YSGG lasers are also successful in coagulating the opened blood vessels and de-epithelizing the gingival pocket [1, 13]. The laser assisted treatment is a better treatment modality compared to the coventional non-surgical periodontal treatment according to several studies [1, 4, 7, 14].

Oral malodor or halitosis is defined as an unpleasent odor originating from the oral cavity or extra-oral sources. The oral region is substantially responsible for oral malodor, which is a result of bacterial putrification of food debris in periodontal pockets or interdental regions [15, 16]. Volatile sulphur compounds (VSC) are sources of bad odor and production of specific oral bacteria such as Treponema denticola, Porphyromonas gingivalis, Prevotella intermedius and Porphyromonas endodontalis, which are promoted in the secluded regions of the oral cavity such as dental cavities and periodontal pockets in which bacterial growth is favoured due to accumalating debris [17, 18].

Oral malodor mostly originates from the oral region and there is a sound link between periodontal disease and oral malodor [19]. Several studies reported that effective periodontal therapy significantly reduced oral malodor [19, 20]. The efficacy of Er,Cr:YSGG laser assisted periodontal therapy on the reduction of oral malodor has not yet been reported. The hypothesis of this study is defined as Er,Cr:YSGG laser assisted periodontal therapy may be more effective in controling periodontal disease related oral malodor compared to conventional periodontal therapy. The aim of this study is to evaluate the efficacy of Er,Cr:YSGG laser assisted periodontal therapy on the reduction of oral malodor and periodontal disease.

## Methods

This study was planned in a double blind fashion. Sixty-seven patients who were referred to the Department of Periodontology, Faculty of Dentistry of Akdeniz University for periodontal therapy due to the complaint of bad breath between October 2014 and December 2014 were enrolled into the study. Inclusion criteria for the study were as follows;

1. Chronic periodontitis with 5 mm or greater pocket depth in at least 2 teeth
2. Older than 18 years old
3. No administration of antibiotics in 6 months period before treatment
4. No history of previous periodontal therapy
5. At least 15 teeth present
6. No systemic diseases
7. No deep carious teeth

One patient refused to be included in the study. Six patients were excluded from the study because they did not meet the inclusion criteria. Sixty patients were included in the study. All patients signed written informed consent forms prior to the study. The study protocol was approved by the local Clinical Research Ethics Commitee with approval number 11/09/2014-46/3 and performed in accordance with the ethical standarts laid down in the 1964 Declaration of Helsinki and its later amendments.

**Fig. 1:**
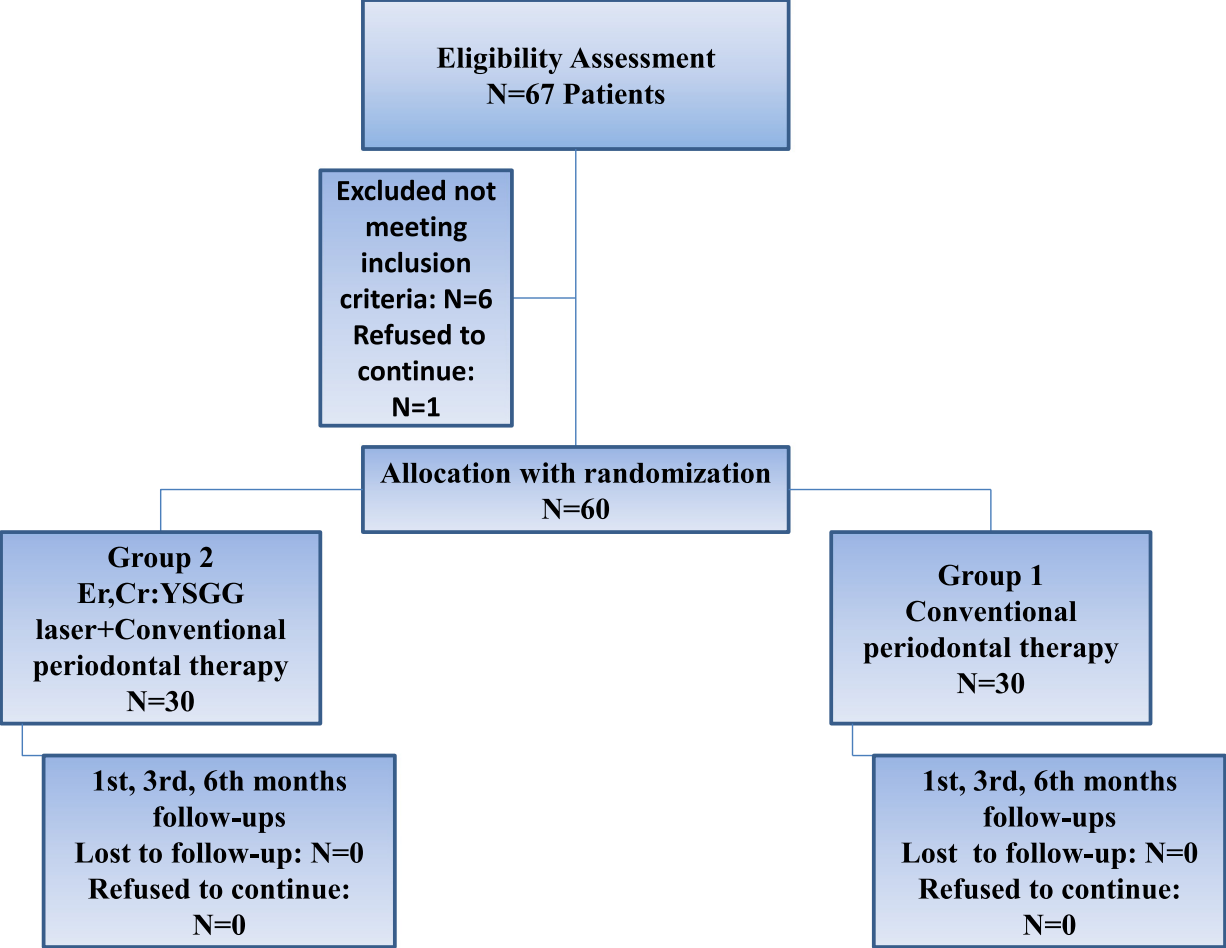
The flow chart of the study protocol

Superficially carious teeth of all selected patients were examined and treated prior to the study. All treatments were performed under local anesthesia. All patients were allocated into group 1 and group 2 by random number generation in Microsoft Excel (Microsoft Corporation, Washington, USA) (Fig. 1). Group 2 comprised conventional scaling and root-plaining followed by immediate Er,Cr:YSGG application. Group 1 comprised conventional scaling root-plaining therapy. Patients did not know to which group they were assigned. Measurements for plaque index (PI), probing depth (PD), clinical attachment level (CAL) and bleeding on probing (BOP) were recorded before treatment (baseline) and after treatment at the 1st, 3rd and 6th months. All teeth with 5 mm or greater pocket depth were treated in the same patient and all measurements of periodontal parameters were performed at six sites per tooth (mesio-buccal, mid-buccal, disto-buccal, mesio-palatal, mid-palatal and disto-palatal) with a Williams 1 mm scaled periodontal probe by one researcher who is blinded to the allocation process. (MH) Plaque index was evaluated with criteria which is proposed by Löe [21]. PD was the distance between the free gingival margin and the deepest point of the pocket. CAL was the distance between the cemento-enamel junction and base of the pocket. BOP was scored as presence and abscence in a period of 30 seconds after probing. BOP presence was determined as a per cent value.

Halitosis scoring was done thru a portable sulfur monitor (Halimeter Interscan, Chatsworth, California, USA) Volatile sulphur compounds were detected with the aid of parts per billion unit (ppb) and ppb monitoring was performed as previously reported [18]. The halimeter was calibrated according to manufacturer’s recommendations. Patients were instructed to hold their breaths and keep their mouths closed for 2 min and not to swallow prior to each measurement. A plastic straw connected to the monitor was inserted approximately 4 cm into the mouth of the patient as they inhaled and exhaled through the nose. Measurement was repeated three times for each patient and the highest value was recorded. All patients were instructed not to use alcohol, smoke, eat garlic or spicy food 1 day before the measurements. Halitosis scoring was done before and 1st, 3rd and 6th months after the ending of all treatment sessions in group 1 and group 2. All measurements were done on 10 a.m. and all patients were instructed to have their breakfast and perform oral hygiene procedures of teeth and tongue brushing 1 h before the measurements.

A Waterlase MD Er,Cr:YSGG laser (Biolase, Irvine, California, USA) with a RFPT 5-14 360° firing tip was used in the study. The firing tip of the laser was used with an angulation of 10° to the root surface and a bottom-up technique in which the laser was applied in a bottom to upward direction with circulation movements in contact with the pocket. Only inside of the pockets were irradiated and each pocket was irradiated once per session. The laser settings were 1.5 W, 30 Hz pulse rate, 11 % air, 20 % H_2_0 and H-Mode (Pulse Duration: 140 us). The laser irradiation was applied three times over a period of 7 days under local anesthesia (40 mg/ml articaine hydrochloride and 0.006 mg adrenaline hydrochloride). In both groups, second session was applied after 48 h after first session and third session was applied on the 7th day after first session. In group 2, laser irradiation was applied immediately after conventional periodontal treatment in session 1 and it was applied alone without conventional periodontal treatment in sessions 2 and 3. In group 1, conventional periodontal treatment was performed in all 3 sessions. For blindness purposes, in group 1, laser firing tip was positioned into the periodontal pockets as in group 2, but not activated.

An ultrasonic scaler and hand instruments (Gracey Curettes, Hu-Friedy, Chicago, IL, US) were used for full mouth subgingival and supragingival scaling and root-plaining. All patients received oral hygiene instructions at the time of the appointment and were given written and verbal recommendations to brush their teeth daily and maintain oral health measures after periodontal therapy.

### Statistical analysis

SPSS version 20.0 (IBM, Chicago, IL, USA) was used for statistical analysis. A Shapiro-Wilk’s test (*p* < 0.05) and a visual inspection of their histograms, normal Q-Q plots and box plots showed that the exam scores were not normally distributed for both groups. Friedman test was used to define the statistical difference level between follow-up time point measurements in the study and control groups seperately. Mann–Whitney-*U* test was used to define the statistical difference level between study and control groups. *P* values <0.05 were accepted as statistically significant.

## Results

The mean age of all patients was 43.7 (±3.1). There were 31 males (51.7 %) and 29 females (48.3 %). All patients included in the study complied with the progress of the study and there were no drop-outs. Healing was uneventful in all patients and no adverse effects such as burning sensation, dentin hypersensitivity or pain were recorded.

The differences of baseline values of all periodontal parameters and ppb values between the study and control groups were not statistically significant. There was statistically significant improvement in all parameters of both group 1 and group 2 from baseline to post-treatment 1st, 3rd and 6th months (*p* < 0.01) (Table 1). Nevertheless, there was a statistically significant reduction in ppb levels at post-treatment 3rd and 6th month in group 2 comparing to group 1 (*p* < 0.05) (Table 1), while there was also a statistically significant reduction in PD at post-treatment 1st month and in BOP at post-treatment 3rd and 6th months in the group 2 comparing to group 1 (*p* < 0.05) (Table 1).

**Table 1:** Mean values and standart derivations of study parameters halitosis (quantitative analysis of VSC volume), probing depth, clinical attachment level, plaque index and bleeding on probing on pre-treatment (baseline), 1st month, 3rd month and 6th month follow-up periods

Follow-up Time Points	Halitosis (ppb)			Probing depth (PD)(mm)			Clinical atachment Level (CAL)(mm)			Plaque index (PI)			Bleeding on probing (BOP)(per cent)		
	Group 1	Group 2	P*	Group 1	Group 2	P*	Group 1	Study	P*	Group 1	Group 2	P*	Group 1	Group 2	P*
Baseline values, mean (SD)	89.7 (13.9)	88.2 (15.2)	0,58	5.3 (1.8)	5.3 (1.8)	0,91	2.9 (0.4)	2.9 (0.6)	0,65	2.5 (0.5)	2.4 (0.5)	0,37	77.7 (7.4)	75.1 (7.2)	0,12
1 months, mean (SD)	63.8 (7.3)	60.6 (8.8)	0.10	2.7 (0.4)	2.3 (0.8)	**<0.05**	2.0 (0.2)	2.0 (0.5)	0.06	1.9 (0.4)	1.8 (0.4)	0.75	48.5 (7.7)	48.5 (9.4)	0.93
3 months, mean (SD)	69.2 (8.7)	54.4 (8.8)	**<0.001**	2.3 (0.6)	2.2 (0.6)	0.78	1.8 (0.4)	1.8 (0.7)	0.76	1.7 (0.4)	1.6 (0.4)	0.57	47.9(6.6)	40.7 (11.0)	**<0.05**
6 months, mean (SD)	68.8 (10.2)	57.1 (12.2)	**<0.05**	2.1 (0.6)	1.9 (0.7)	0.13	1.9 (0.4)	1.8 (0.5)	0.20	1.5(0.5)	1.5 (0.5)	0.71	41.6 (8.6)	37.8 (7.7)	**<0.05**
P**	**<0.01**	**<0.01**	**-**	**<0.01**	**<0.01**	**-**	**<0.01**	**<0.01**	**-**	**<0.01**	**<0.01**	**-**	**<0.01**	**<0.01**	-

Statistically significant data was shown in the bold emphasis

*p values in the columns represents significance between study and control groups (*p* < 0.05)

**p value in the rows represents significance between measurements on time periods in study and control groups separately (*p* < 0.05)

## Discussion

Laser-assisted dentistry is a recent emerging trend. Dental lasers are frequently used in oral surgical procedures as well as restorative dentistry and prosthodontics. Er,Cr:YSGG lasers have been used since the last decade in dentistry and reported to have provided better periodontal tissue regeneration than that of conventional non-surgical periodontal therapy [5, 6]. Kelbauskiene et al. [5]. reported that a combination of Er,Cr:YSGG laser and conventional scaling and root plaining had better results compared to scaling root-plaining alone in terms of attachment level restoration. Other types of high intensity lasers such as Er:YAG and Nd:YAG lasers have been recently used in a way similar to Er,Cr:YSGG in the periodontal therapy [22, 23]. Er:YAG lasers are securely used as an alternative to non-surgical periodontal therapy or as an adjunct for pocket treatment [24, 25]. However, there is few evidence showing the superiority of ER:YAG lasers to others [23]. Quadri et al. [26] reported that SRP in combination with a single application of Nd:YAG laser significantly promotes periodontal healing compared to SRP alone. However, diode and Nd:YAG lasers are reported to have a profound complication of excessive heating of surrounding tissues [27]. It is reported that the damage of the termal side effects is prominently reduced in Er,Cr:YSGG laser and it can be safely and effectively used in non-surgical periodontal therapy [4]. Although there have been certain advantages of adjunctive use of lasers in periodontal therapy, recent studies suggest that the benefits of adjunctive lasers are questionable compared to other periodontal therapy methods. In a meta-analysis conducted by Smiley et al. [28], it is reported that photodynamic laser therapy with diode laser used as an adjunct to SRP is considered beneficial with a moderate level of certainty. In addition, Birang et al. [29] reported that adjunctive laser therapy demonstrated minimal benefits compared to adjunctive photodynamic therapy in the treatment of chronic periodontitis.

In the current study, there was statistically significant improvement in all periodontal parameters in both group 1 and group 2 at all time points (*p* < 0.01). However, the difference between group 1 and group 2 was not significant in all time periods for periodontal parameters, especially in PI and CAL. This finding is not consistent with the literature [1, 5, 7]. Kelbauskiene et al. [5] reported that periodontal parameters of PD, BOP and PI in patients treated with Er,Cr:YSGG laser assisted periodontal therapy are significantly improved compared to patients treated with SRP alone. Gupta et al. [4] reported no significant difference between CAL and PI levels of two groups consisting of Er,Cr:YSGG assisted conventional periodontal therapy and open flap debridement. In the current study, there was a statistically significant decrease in PD at 1st month after treatment in the laser group. However, there was no statistically significant difference between PD values of group 1 and group 2 at the 3rd and 6th month after treatment. This finding is consistent with the study of Gutknecht et al. [1], who reported that laser irradiation did not have a significant effect on reducing pocket depth compared to non-laser conventional treatment. However, they concluded that the antibacterial effect of laser treatment was very effective. Similar to PD, a marked reduction of BOP scores was observed in the laser treatment group and this is in accordance with the literature findings [4, 5].

Halitosis is bad odor emanating from the oral region and majorly originates from the oral cavity [20]. Pham et al. [19] reported that oral malodor is directly correlated with periodontal disease. Oral malodor is evaluated by two main methods: one is a subjective and the other an objective evaluation method [30, 31]. Subjective evaluation is also called organoleptic evaluation which is performed by the researcher by means of inhaling the breath of the patient directly. Objective methods such as gas chromotography and quantitative analysis of VSCs are useful to obtain quantitative data regarding oral malodor. Although an organolaeptic method is cost-effective and easier to perform, it has a disadvantage of being subjective. In the current study, the objective method of quantitative measurement of VSC was prefered because it provided a numeric value. Yaegaki & Sanada [32] reported that VSC levels are directly related with periodontal bleeding. However, this finding does not support the idea that all patients with periodontitis will certainly have oral malodor or vice versa. In the current study, VSC levels showed a dramatic decrease in patients treated with Er,Cr:YSGG laser compared to conventional periodontal therapy, revealing that conventional periodontal therapy with the asisstance of Er,Cr:YSGG laser may be more effective than conventional periodontal therapy in reducing oral malodor. Similar to this study, in the study conducted by Silveira et al. [33] in which a sample size of 27 patients were included, it is reported that supragingival plaque control reduced halitosis in patients with periodontitis. In the study of Kara et al. [18], who compared the efficacy of a Nd: YAG laser on reducing oral malodor to conventional periodontal therapy in a population of 60 patients, it is suggested that Nd:YAG laser was superior in reducing oral malodor compared to conventional periodontal therapy.

Tongue coating is defined as one of the primary sites of oral malodor [34]. In the current study, patients were encouraged to cleanse their tongue by brushing the dorsal region of the tongue. However, a score for tongue coating was not recorded. This is one of the limitations of this study. Additionally, oral hygiene education regarding tongue cleansing might have contributed to the reduction of oral malodor.

The effectiveness of laser therapy on oral malodor has been reported in several studies in the literature [18, 35]. Kara et al. [18] reported that Nd:YAG lasers had an adjunctive role in reducing oral malodor compared to conventional periodontal therapy. They also concluded that oral malodor and periodontal disease levels are directly related. In the study of Lopez et al. [35], photodynamic therapy was applied to the dorsum of the tongue of teenager patients suffering from oral malodor and was observed to be effective in reducing oral malodor. However, the success of adjunctive use of diode laser with conventional periodontal therapy is emphasized to be questionable in recent studies [36, 37].

In the present study, a significant decrease in BOP and halitosis was observed in 3rd and 6th months in patients treated with Er,Cr:YSGG assisted conventional periodontal therapy. The significant change in these parameters brings up the assumption of the reduction in periodontal disease related halitosis may be associated with the reduction of BOP. VSCs are mainly produced from bacterial colonies residing in the periodontal pocket [38]. Thus, reduction in halitosis may be explained by the elimination of the VSC producing bacteria in the periodontal pocket. However, a more definite examination and a separate study protocol are needed to expose the true relationship between BOP and halitosis.

## Conclusion

Although there is an ongoing debate about the benefits of the usage of adjunctive laser therapy with conventional periodontal treatment, the present study confirms that Er,Cr:YSGG assisted periodontal therapy improves periodontal parameters of PD and BOP in chronic periodontitis and reduces periodontal disease related oral malodor more effectively than conventional non-surgical periodontal therapy. The current study to the best of our knowledge is the first to evaluate the efficacy of Er,Cr:YSGG laser on halitosis. The study population should be expanded in further studies in order to achieve more definite results.

## Abbreviations

BOP: bleeding on probing
CAL: clinical attachment level
PD: probing depth
PI: plaque index
VSC: volatile sulphur compounds
